# (11*R*,11a*S*)-11-Hydr­oxy-1,5,11,11a-tetra­hydro-1-benzothieno[2,3-*f*]indolizin-3(2*H*)-one

**DOI:** 10.1107/S1600536808015456

**Published:** 2008-05-30

**Authors:** Ľubomír Švorc, Viktor Vrábel, Jozef Kožíšek, Štefan Marchalín, Peter Šafář

**Affiliations:** a Institute of Analytical Chemistry, Faculty of Chemical and Food Technology, Slovak Technical University, Radlinského 9, SK-812 37 Bratislava, Slovak Republic; b Institute of Physical Chemistry and Chemical Physics, Faculty of Chemical and Food Technology, Slovak Technical University, Radlinského 9, SK-812 37 Bratislava, Slovak Republic; c Institute of Organic Chemistry, Catalysis and Petrochemistry, Faculty of Chemical and Food Technology, Slovak Technical University, Radlinského 9, SK-812 37 Bratislava, Slovak Republic

## Abstract

The absolute configuration of the title compound, C_14_H_13_NO_2_S, was assigned from the synthesis and confirmed by the structure determination. The central six-membered ring of the indolizine system adopts an envelope conformation, the greatest deviation from the mean plane of the ring being 0.459 (2) Å for the N atom. The benzothieno system is planar [mean deviation = 0.009 (2) Å]. In the crystal structure, mol­ecules form chains parallel to the *b* axis *via* inter­molecular O—H⋯O hydrogen bonds.

## Related literature

For related literature, see: Campagna *et al.* (1990 ▶); Camus *et al.* (2000 ▶); Gubin *et al.* (1992 ▶); Gupta *et al.* (2003 ▶); Malonne *et al.* (1998 ▶); Medda *et al.* (2003 ▶); Mitsumori *et al.* (2004 ▶); Nardelli (1983 ▶); Ostrander *et al.* (1988 ▶); Pearson & Guo (2001 ▶); Ruprecht *et al.* (1989 ▶); Sonnet *et al.* (2000 ▶); Teklu *et al.* (2005 ▶); Vlahovici *et al.* (2002 ▶); Vrábel *et al.* (2004 ▶); Šafář *et al.* (2008 ▶).
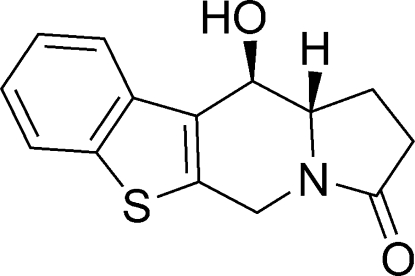


## Experimental

### 

#### Crystal data

C_14_H_13_NO_2_S
*M* *_r_* = 259.31
Orthorhombic, 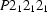

*a* = 7.6614 (1) Å
*b* = 11.7733 (2) Å
*c* = 13.0736 (2) Å
*V* = 1179.24 (3) Å^3^
*Z* = 4
Mo *K*α radiation
μ = 0.27 mm^−1^
*T* = 298 (2) K
0.50 × 0.30 × 0.28 mm

#### Data collection

Oxford Diffraction Gemini R CCD diffractometer
Absorption correction: analytical (Clark & Reid, 1995 ▶) *T*_min_ = 0.867, *T*_max_ = 0.941
32596 measured reflections
3149 independent reflections
2599 reflections with *I* > 2σ(*I*)
*R*_int_ = 0.018

#### Refinement

*R*[*F* ^2^ > 2σ(*F* ^2^)] = 0.029
*wR*(*F* ^2^) = 0.076
*S* = 1.04
3149 reflections
165 parameters
H-atom parameters constrained
Δρ_max_ = 0.21 e Å^−3^
Δρ_min_ = −0.17 e Å^−3^
Absolute structure: Flack (1983 ▶), 1259 Friedel pairs
Flack parameter: 0.01 (6)

### 

Data collection: *CrysAlis CCD* (Oxford Diffraction, 2006 ▶); cell refinement: *CrysAlis RED* (Oxford Diffraction, 2006 ▶); data reduction: *CrysAlis RED*; program(s) used to solve structure: *SHELXS97* (Sheldrick, 2008 ▶); program(s) used to refine structure: *SHELXL97* (Sheldrick, 2008 ▶); molecular graphics: *DIAMOND* (Brandenburg, 2001 ▶); software used to prepare material for publication: *enCIFer* (Allen *et al.*, 2004 ▶).

## Supplementary Material

Crystal structure: contains datablocks I. DOI: 10.1107/S1600536808015456/bq2077sup1.cif

Structure factors: contains datablocks I. DOI: 10.1107/S1600536808015456/bq2077Isup2.hkl

Additional supplementary materials:  crystallographic information; 3D view; checkCIF report

## Figures and Tables

**Table 1:** Hydrogen-bond geometry (Å, °)

*D*—H⋯*A*	*D*—H	H⋯*A*	*D*⋯*A*	*D*—H⋯*A*
O2—H2⋯O1^i^	0.82	2.00	2.822 (2)	174

Symmetry code: (i) 
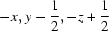
.

**Fig. 1.**
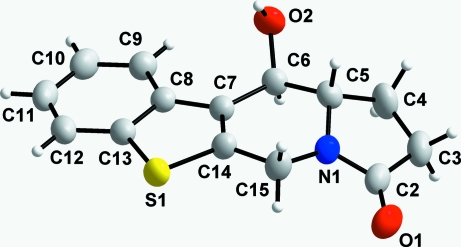
Molecular structure of (I) with the atomic numbering scheme. Displacement ellipsoids are drawn at the 50% probability level.

**Fig. 2.**
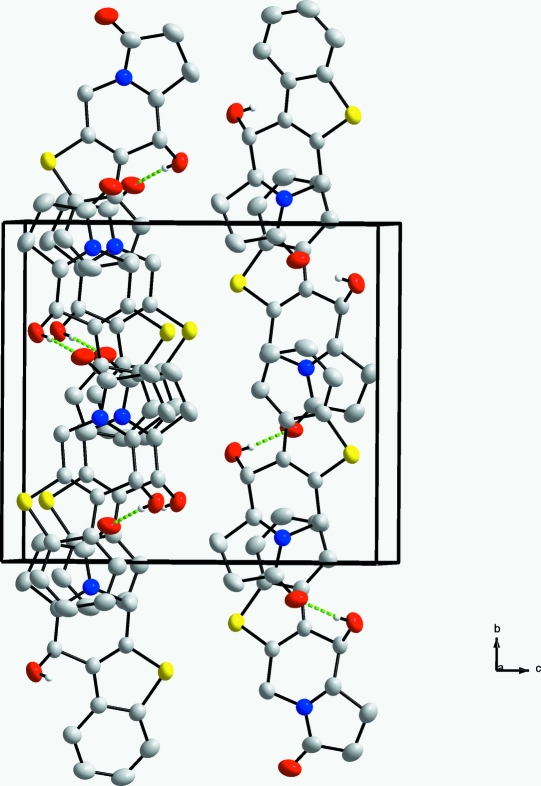
A packing diagram of the compound (I). Dashed lines indicate hydrogen bonds.

## supplementary crystallographic information

### Comment

Indolizine derivatives have been found to possess a variety of biological activities such as antiinflammatory (Malonne *et al.*, 1998), antiviral (Medda *et al.*, 2003), aromatase inhibitory (Sonnet *et al.*, 2000), analgestic (Campagna *et al.*, 1990) and antitumor (Pearson & Guo, 2001) activities. They have also shown to be calcium entry blockers (Gupta *et al.*, 2003) and potent antioxidants inhibiting lipid peroxidation *in vitro* (Teklu *et al.*, 2005). As such, indolizines are important synthetic targets in view of developing new pharmaceuticals for the treatment of cancer (Ostrander *et al.*, 1988), cardiovascular diseases (Gubin *et al.*, 1992) and HIV infections (Ruprecht *et al.*, 1989). Polycyclic indolizine derivatives have been found to have high-efficiency long-wavelength fluorescence quantum yield (Vlahovici *et al.*, 2002). The synthesis of polycyclic indolizine derivatives has recently attracted much research interest in the search for new opto-electric materials (Mitsumori *et al.*, 2004). As part of our recent efforts to synthesize novel polycyclic indolizine derivative, we report here the synthesis and molecular and crystal structure of the title compound, (I) (Fig. 1). The absolute configuration has been established without ambiguity from the anomalous dispersion of the S atom [absolute structure parameter 0.01 (6) (Flack, 1983)] and assigned consistent with the starting material. The expected stereochemistry of atoms C5 and C6 was confirmed as S and *R*, respectively (Fig. 1). The central N-heterocyclic ring is not planar and adopts an envelope conformation (Nardelli, 1983). A calculation of least-squares planes shows that this ring is puckered in such a manner that the five atoms C5, C6, C7, C14 and C15 are planar to within 0.061 (3) Å, while atom N1 is displaced from this plane with out-of-plane displacement of 0.459 (2) Å. The pyrrolidin-2-one ring is distorted towards a flat-envelope conformation, with atom C5 on the flap. Atom C5 is 0.291 (2)Å from the mean plane defined by atoms N1, C2, C3 and C4. The molecule as a whole is nonplanar but consist of two approximately planar segments, C5, C6, C7, C8, C9, C10, C11, C12, C13, S1, C14, C15 [r.m.s. deviation 0.086 (2) Å] and N1, C2, O1, C3, C4 [r.m.s. deviation 0.046 (3) Å] with dihedral angle 27.0 (1)°. Atom N1 is *sp*^2^-hybridized, as evidenced by the sum of the valence angles around it (358.8°). These data are consistent with conjugation of the lone-pair electrons on N1 with the adjacent carbonyl, similar to what is observed for amides. Intermolecular O—H···O hydrogen bonds link the molecules of (I) into infinite chains, which run parallel to the *b* axis (Fig. 2 and Table 2) and help to stabilize the crystal structure of the compound. The bond lengths of the carbonyl group C2=O1 is 1.221 (2)Å somewhat longer than typical carbonyl bonds. This may be due to the fact that atom O1 participates in intermolecular hydrogen bond. The bond lengths and angles in the indolizine ring system are comparable with those in related structures (Camus, *et al.*, 2000; Vrábel, *et al.*, 2004).

### Experimental

The title compound (11*R*,11aS)-11-hydroxy-1,5,11,11*a*-tetrahydro[1] benzothieno[2,3-*f*]indolizin-3(2*H*)-one was prepared according literature procedures of Šafář, *et al.* (2008).

### Refinement

All H atoms were placed in geometrically idealized positions and constrained to ride on their parent atoms, with C—H distances in the range 0.93 - 0.98Å and O—H distance 0.85Å and *U*_iso_ set at 1.2*U*_eq_ of the parent atom. The absolute configuration has been determined. The number of Friedel pairs is 1259.

### Crystal data

**Table:**

C_14_H_13_NO_2_S	*F*_000_ = 544
*M**_r_* = 259.31	*D*_x_ = 1.461 Mg m^−^^3^
Orthorhombic, *P*2_1_2_1_2_1_	Mo *K*α radiation λ = 0.71073 Å
Hall symbol: P 2ac 2ab	Cell parameters from 22009 reflections
*a* = 7.6614 (1) Å	θ = 3.1–26.4º
*b* = 11.7733 (2) Å	µ = 0.27 mm^−^^1^
*c* = 13.0736 (2) Å	*T* = 298 (2) K
*V* = 1179.24 (3) Å^3^	Block, white
*Z* = 4	0.50 × 0.30 × 0.28 mm

### Data collection

**Table:**

Oxford Diffraction Gemini R CCD diffractometer	3149 independent reflections
Radiation source: fine-focus sealed tube	2599 reflections with *I* > 2σ(*I*)
Monochromator: graphite	*R*_int_ = 0.019
Detector resolution: 10.4340 pixels mm^-1^	θ_max_ = 26.6º
*T* = 298(2) K	θ_min_ = 3.1º
Rotation method data acquisition using ω and φ scans	*h* = −10→10
Absorption correction: analytical(Clark & Reid, 1995)	*k* = −16→15
*T*_min_ = 0.867, *T*_max_ = 0.941	*l* = −17→17
32596 measured reflections	

### Refinement

**Table:**

Refinement on *F*^2^	H-atom parameters constrained
Least-squares matrix: full	*w* = 1/[σ^2^(*F*_o_^2^) + (0.0357*P*)^2^ + 0.2112*P*] where *P* = (*F*_o_^2^ + 2*F*_c_^2^)/3
*R*[*F*^2^ > 2σ(*F*^2^)] = 0.029	(Δ/σ)_max_ < 0.001
*wR*(*F*^2^) = 0.076	Δρ_max_ = 0.21 e Å^−^^3^
*S* = 1.04	Δρ_min_ = −0.17 e Å^−^^3^
3149 reflections	Extinction correction: SHELXL97 (Sheldrick, 2008), Fc^*^=kFc[1+0.001xFc^2^λ^3^/sin(2θ)]^-1/4^
165 parameters	Extinction coefficient: 0.0198 (16)
Primary atom site location: structure-invariant direct methods	Absolute structure: Flack (1983), 1259 Friedel pairs
Secondary atom site location: difference Fourier map	Flack parameter: 0.01 (6)
Hydrogen site location: inferred from neighbouring sites	

### Special details

**Table:**

Experimental. face-indexed (*CrysAlis RED*; Oxford Diffraction, 2006)
Geometry. All e.s.d.'s (except the e.s.d. in the dihedral angle between two l.s. planes) are estimated using the full covariance matrix. The cell e.s.d.'s are taken into account individually in the estimation of e.s.d.'s in distances, angles and torsion angles; correlations between e.s.d.'s in cell parameters are only used when they are defined by crystal symmetry. An approximate (isotropic) treatment of cell e.s.d.'s is used for estimating e.s.d.'s involving l.s. planes.
Refinement. Refinement of *F*^2^ against ALL reflections. The weighted *R*-factor *wR* and goodness of fit *S* are based on *F*^2^, conventional *R*-factors *R* are based on *F*, with *F* set to zero for negative *F*^2^. The threshold expression of *F*^2^ > σ(*F*^2^) is used only for calculating *R*-factors(gt) *etc*. and is not relevant to the choice of reflections for refinement. *R*-factors based on *F*^2^ are statistically about twice as large as those based on *F*, and *R*- factors based on ALL data will be even larger.

### Fractional atomic coordinates and isotropic or equivalent isotropic displacement parameters (Å^2^)

**Table:**

	*x*	*y*	*z*	*U*_iso_*/*U*_eq_	
C2	0.1944 (2)	1.03675 (13)	0.20976 (13)	0.0455 (3)	
C3	0.1497 (3)	1.05817 (14)	0.09913 (15)	0.0557 (4)	
H3A	0.0504	1.1089	0.0935	0.067*	
H3B	0.2480	1.0916	0.0634	0.067*	
C4	0.1063 (3)	0.94225 (15)	0.05531 (14)	0.0582 (5)	
H4A	0.2014	0.9147	0.0131	0.070*	
H4B	0.0012	0.9459	0.0140	0.070*	
C5	0.0789 (2)	0.86434 (13)	0.14788 (12)	0.0404 (3)	
H5	−0.0462	0.8611	0.1634	0.048*	
C6	0.14675 (19)	0.74306 (12)	0.13407 (10)	0.0378 (3)	
H6	0.2456	0.7462	0.0866	0.045*	
C7	0.21221 (18)	0.69361 (12)	0.23315 (10)	0.0354 (3)	
C8	0.27062 (19)	0.57799 (12)	0.24570 (11)	0.0372 (3)	
C9	0.2764 (2)	0.48925 (13)	0.17477 (12)	0.0440 (4)	
H9	0.2386	0.5010	0.1080	0.053*	
C10	0.3382 (2)	0.38453 (14)	0.20415 (15)	0.0509 (4)	
H10	0.3420	0.3257	0.1567	0.061*	
C11	0.3952 (2)	0.36526 (14)	0.30371 (15)	0.0517 (4)	
H11	0.4352	0.2935	0.3221	0.062*	
C12	0.3932 (2)	0.45092 (14)	0.37530 (14)	0.0475 (4)	
H12	0.4322	0.4383	0.4417	0.057*	
C13	0.33100 (19)	0.55722 (12)	0.34551 (11)	0.0397 (3)	
C14	0.23124 (19)	0.75541 (12)	0.31979 (10)	0.0382 (3)	
C15	0.1883 (3)	0.87867 (12)	0.33040 (11)	0.0455 (3)	
H15A	0.0815	0.8878	0.3694	0.055*	
H15B	0.2816	0.9177	0.3662	0.055*	
N1	0.16682 (18)	0.92626 (10)	0.22933 (10)	0.0413 (3)	
O1	0.24479 (19)	1.10666 (10)	0.27209 (11)	0.0614 (3)	
O2	0.01482 (15)	0.67549 (10)	0.08796 (8)	0.0503 (3)	
H2	−0.0601	0.6601	0.1306	0.076*	
S1	0.31793 (6)	0.67853 (3)	0.42095 (3)	0.04613 (11)	

### Atomic displacement parameters (Å^2^)

**Table:**

	*U*^11^	*U*^22^	*U*^33^	*U*^12^	*U*^13^	*U*^23^
C2	0.0448 (8)	0.0367 (7)	0.0551 (9)	0.0070 (7)	0.0090 (8)	0.0030 (7)
C3	0.0607 (10)	0.0477 (8)	0.0586 (10)	0.0112 (8)	0.0067 (8)	0.0151 (8)
C4	0.0766 (12)	0.0550 (10)	0.0431 (9)	0.0100 (9)	−0.0054 (8)	0.0113 (7)
C5	0.0390 (7)	0.0453 (8)	0.0369 (7)	0.0062 (6)	0.0006 (6)	0.0016 (6)
C6	0.0422 (7)	0.0422 (7)	0.0290 (6)	0.0025 (6)	0.0025 (5)	0.0004 (5)
C7	0.0382 (7)	0.0377 (7)	0.0302 (6)	−0.0005 (6)	0.0023 (5)	0.0020 (5)
C8	0.0360 (7)	0.0384 (7)	0.0371 (7)	−0.0011 (5)	0.0027 (6)	0.0025 (5)
C9	0.0455 (9)	0.0424 (8)	0.0440 (8)	0.0016 (6)	0.0044 (7)	−0.0026 (6)
C10	0.0487 (9)	0.0423 (8)	0.0618 (10)	0.0047 (7)	0.0083 (8)	−0.0046 (7)
C11	0.0423 (8)	0.0401 (8)	0.0727 (11)	0.0050 (7)	0.0049 (8)	0.0099 (8)
C12	0.0417 (8)	0.0485 (9)	0.0523 (9)	0.0003 (7)	−0.0017 (7)	0.0131 (8)
C13	0.0393 (7)	0.0411 (7)	0.0389 (7)	−0.0018 (6)	0.0016 (6)	0.0050 (6)
C14	0.0466 (8)	0.0370 (7)	0.0309 (6)	−0.0023 (6)	0.0010 (6)	0.0016 (5)
C15	0.0620 (10)	0.0398 (7)	0.0348 (7)	0.0024 (7)	0.0027 (7)	−0.0025 (6)
N1	0.0494 (7)	0.0368 (6)	0.0377 (6)	0.0035 (5)	0.0022 (6)	0.0005 (5)
O1	0.0725 (8)	0.0396 (6)	0.0720 (8)	0.0007 (6)	0.0049 (7)	−0.0072 (6)
O2	0.0591 (7)	0.0554 (6)	0.0365 (5)	−0.0017 (6)	−0.0071 (5)	−0.0085 (6)
S1	0.0593 (2)	0.04554 (19)	0.03357 (17)	−0.00295 (18)	−0.00694 (17)	0.00349 (16)

### Geometric parameters (Å, °)

**Table:**

C2—O1	1.221 (2)	C8—C9	1.398 (2)
C2—N1	1.3426 (19)	C8—C13	1.406 (2)
C2—C3	1.508 (2)	C9—C10	1.375 (2)
C3—C4	1.517 (3)	C9—H9	0.9300
C3—H3A	0.9700	C10—C11	1.392 (3)
C3—H3B	0.9700	C10—H10	0.9300
C4—C5	1.533 (2)	C11—C12	1.376 (3)
C4—H4A	0.9700	C11—H11	0.9300
C4—H4B	0.9700	C12—C13	1.395 (2)
C5—N1	1.456 (2)	C12—H12	0.9300
C5—C6	1.530 (2)	C13—S1	1.7385 (15)
C5—H5	0.9800	C14—C15	1.494 (2)
C6—O2	1.4206 (18)	C14—S1	1.7348 (14)
C6—C7	1.5061 (18)	C15—N1	1.4445 (18)
C6—H6	0.9800	C15—H15A	0.9700
C7—C14	1.3541 (19)	C15—H15B	0.9700
C7—C8	1.442 (2)	O2—H2	0.8200
			
O1—C2—N1	125.15 (16)	C9—C8—C7	129.79 (13)
O1—C2—C3	126.83 (15)	C13—C8—C7	111.83 (12)
N1—C2—C3	108.01 (14)	C10—C9—C8	119.72 (15)
C4—C3—C2	105.17 (13)	C10—C9—H9	120.1
C4—C3—H3A	110.7	C8—C9—H9	120.1
C2—C3—H3A	110.7	C9—C10—C11	121.02 (16)
C4—C3—H3B	110.7	C9—C10—H10	119.5
C2—C3—H3B	110.7	C11—C10—H10	119.5
H3A—C3—H3B	108.8	C12—C11—C10	120.88 (15)
C3—C4—C5	105.67 (14)	C12—C11—H11	119.6
C3—C4—H4A	110.6	C10—C11—H11	119.6
C5—C4—H4A	110.6	C11—C12—C13	118.14 (16)
C3—C4—H4B	110.6	C11—C12—H12	120.9
C5—C4—H4B	110.6	C13—C12—H12	120.9
H4A—C4—H4B	108.7	C12—C13—C8	121.85 (14)
N1—C5—C6	113.35 (12)	C12—C13—S1	126.76 (12)
N1—C5—C4	102.39 (13)	C8—C13—S1	111.39 (11)
C6—C5—C4	114.74 (13)	C7—C14—C15	125.15 (13)
N1—C5—H5	108.7	C7—C14—S1	113.49 (11)
C6—C5—H5	108.7	C15—C14—S1	121.35 (11)
C4—C5—H5	108.7	N1—C15—C14	108.47 (12)
O2—C6—C7	112.67 (12)	N1—C15—H15A	110.0
O2—C6—C5	109.32 (12)	C14—C15—H15A	110.0
C7—C6—C5	111.86 (12)	N1—C15—H15B	110.0
O2—C6—H6	107.6	C14—C15—H15B	110.0
C7—C6—H6	107.6	H15A—C15—H15B	108.4
C5—C6—H6	107.6	C2—N1—C15	122.16 (13)
C14—C7—C8	112.24 (12)	C2—N1—C5	114.75 (13)
C14—C7—C6	123.20 (12)	C15—N1—C5	121.84 (12)
C8—C7—C6	124.47 (12)	C6—O2—H2	109.5
C9—C8—C13	118.38 (13)	C14—S1—C13	91.04 (7)
			
O1—C2—C3—C4	176.43 (17)	C9—C8—C13—C12	−1.0 (2)
N1—C2—C3—C4	−4.46 (19)	C7—C8—C13—C12	179.72 (14)
C2—C3—C4—C5	15.15 (19)	C9—C8—C13—S1	178.57 (12)
C3—C4—C5—N1	−19.57 (18)	C7—C8—C13—S1	−0.73 (16)
C3—C4—C5—C6	−142.81 (14)	C8—C7—C14—C15	177.89 (15)
N1—C5—C6—O2	155.60 (12)	C6—C7—C14—C15	1.1 (2)
C4—C5—C6—O2	−87.26 (15)	C8—C7—C14—S1	−0.54 (16)
N1—C5—C6—C7	30.10 (17)	C6—C7—C14—S1	−177.34 (11)
C4—C5—C6—C7	147.24 (14)	C7—C14—C15—N1	−14.4 (2)
O2—C6—C7—C14	−132.48 (14)	S1—C14—C15—N1	163.94 (11)
C5—C6—C7—C14	−8.85 (19)	O1—C2—N1—C15	2.6 (3)
O2—C6—C7—C8	51.11 (18)	C3—C2—N1—C15	−176.58 (15)
C5—C6—C7—C8	174.74 (13)	O1—C2—N1—C5	169.96 (16)
C14—C7—C8—C9	−178.38 (16)	C3—C2—N1—C5	−9.17 (18)
C6—C7—C8—C9	−1.6 (2)	C14—C15—N1—C2	−153.70 (14)
C14—C7—C8—C13	0.81 (18)	C14—C15—N1—C5	39.8 (2)
C6—C7—C8—C13	177.57 (13)	C6—C5—N1—C2	142.58 (13)
C13—C8—C9—C10	0.8 (2)	C4—C5—N1—C2	18.41 (17)
C7—C8—C9—C10	179.94 (16)	C6—C5—N1—C15	−49.97 (19)
C8—C9—C10—C11	0.1 (3)	C4—C5—N1—C15	−174.13 (15)
C9—C10—C11—C12	−0.8 (3)	C7—C14—S1—C13	0.10 (12)
C10—C11—C12—C13	0.6 (2)	C15—C14—S1—C13	−178.39 (13)
C11—C12—C13—C8	0.3 (2)	C12—C13—S1—C14	179.90 (14)
C11—C12—C13—S1	−179.18 (12)	C8—C13—S1—C14	0.37 (12)

### Hydrogen-bond geometry (Å, °)

**Table:**

*D*—H···*A*	*D*—H	H···*A*	*D*···*A*	*D*—H···*A*
O2—H2···O1^i^	0.82	2.00	2.822 (2)	174

Symmetry codes: (i) −*x*, *y*−1/2, −*z*+1/2.
